# Tumor suppressor p53 regulates insulin receptor (*INSR*) gene expression via direct binding to the *INSR* promoter

**DOI:** 10.18632/oncotarget.27645

**Published:** 2020-06-23

**Authors:** Rive Sarfstein, Haim Werner

**Affiliations:** ^1^Department of Human Molecular Genetics and Biochemistry, Sackler School of Medicine, Tel Aviv University, Tel Aviv 69978, Israel; ^2^Yoran Institute for Human Genome Research, Tel Aviv University, Tel Aviv 69978, Israel

**Keywords:** insulin, insulin-like growth factor-1 (IGF1), insulin receptor, IGF1 receptor, p53

## Abstract

A significant volume of clinical and epidemiological data provides support to the concept that insulin and the insulin receptor (INSR) have an important role in breast cancer. Tumor suppressor p53 is the most frequently mutated molecule in human cancer. The present study was aimed at evaluating the hypothesis that p53 governs the expression and activation of the *INSR* gene in breast cancer cells. In addition, the study was designed to investigate the mechanism of action of p53 in the context of *INSR* gene regulation. The availability of MCF7 breast cancer-derived cell lines with specific disruption of either the insulin-like growth factor-1 receptor (IGF1R) or INSR allowed us to address the impact of the IGF1R and INSR pathways on p53 expression. Data indicate that the *INSR* gene constitutes a target for p53 action. Wild-type p53 stimulated *INSR* promoter activity in control cells while disruption of endogenous IGF1R or INSR led to inhibition of promoter activity by p53. Mutant p53 strongly stimulated *INSR* promoter. Furthermore, p53 directly binds to the *INSR* promoter in cells with a disrupted IGF1R. Combined, our results identified complex functional and physical interactions between p53 and the INSR pathway. The implications of the p53-INSR interplay in breast cancer needs to be further investigated.

## INTRODUCTION

The insulin/insulin-like growth factors (IGFs) create an hormonal network responsible for the regulation of important physiological events throughout life [1–3]. The biological information conveyed by this complex system governs multiple metabolic, endocrine, nutritional and growth processes [4–7]. In addition, this growth factor system plays key developmental roles at each stage of life, from early *in utero* phases until old age [8, 9]. The insulin/IGF family includes three ligands [insulin, IGF1, IGF2], three cell-surface receptors [insulin receptor (INSR), IGF1 receptor (IGF1R) and IGF2 receptor (IGF2R)], and six IGF-binding proteins (IGFBP1-6) [10–13]. Moreover, a number of non-classical insulin-like molecules have been identified in recent years, including the insulin receptor-related receptor (IRR), insulin-IGF1 hybrid receptor and a number of IGFBP-related proteins [14, 15]. The physiological roles of these new members, for the most part, are yet to be elucidated.

The classical view that emerged following the cloning and characterization of the *INSR* and *IGF1R* genes in the mid-1980s postulated that activation of INSR by insulin leads, predominantly, to metabolic activities [11, 16–19]. On the other hand, activation of IGF1R by IGF1 or IGF2 (depending on the specific organ involved and developmental stage) leads primarily to growth and differentiation events. Despite the fact that this model was based on vast experimental and clinical evidence, it is clear today that this dogmatic representation of *‘insulin/IGF1 labor division’* amounts to an oversimplification of facts [20]. One of the cardinal questions still in need of a biologically plausible rationalization is why the INSR and IGF1R, even though they share the majority of their downstream cytoplasmic targets and signaling pathways, are yet responsible for mediating distinct physiological and pathological activities.

In recent years the literature has provided ample evidence of a cross-talk between the various ligands and receptors of the insulin/IGF family [21, 22]. Both hormones are capable of activating (phosphorylating) the opposite receptor, leading to atypical mechanistic events. These cross-talk events, which are generally achieved at high doses of the ligand (usually one order of magnitude higher than the concentrations required by the high affinity-binding ligands to activate their cognate receptor) may result in activation of INSR by IGF1 (with ensuing metabolic activities). In contrast, activation of IGF1R by insulin may lead to growth events.

P53 is a transcription factor that usually accumulates in the cell in response to various insults, most notably DNA damage [23, 24]. In agreement with its tumor suppressor role, hyperphosphorylated p53 arrests cell cycle progression at the G1 phase, hence enabling damaged DNA to be repaired before the replicative phase [25]. Tumor suppressor p53 is the most frequently mutated molecule in human cancer [26]. In previous studies, we provided evidence that wild-type p53 negatively regulates *IGF1R* gene expression [27, 28]. Co-expression experiments along with *in vitro* transcription assays demonstrated that the mechanism of action of wild-type p53 involves transcriptional suppression of the *IGF1R* gene. In contrast, and consistent with their oncogenic role, tumor-derived mutant forms of p53 enhanced *IGF1R* gene expression [27].

Given the emerging evidence of proliferative and potentially anti-apoptotic actions of INSR, we investigated in the present paper the regulation of the *INSR* gene promoter by wild-type and mutant p53 in breast cancer cells. Using cells with specific disruption of the INSR (INSR-KD) or IGF1R (IGF1R-KD), we also assessed the effect of each one of these signaling pathways on p53 expression and activity. Our data indicate that: (1) activation of p53 is negatively regulated by IGF1R, as indicated by the augmented phosphorylation of p53 in IGF1R-KD cells; (2) p53 directly binds to the *INSR* promoter region in cells with a disrupted IGF1R; (3) wild-type p53 represses *INSR* promoter activity in IGF1R-KD and INSR-KD cells, while enhancing promoter activity in control cells; (4) mutant p53 stimulates *INSR* promoter activity in breast cancer cells. In conclusion, data is consistent with complex interactions between p53 and the IGF1/insulin signaling pathways in regulation of *INSR* gene transcription. The impact of these interactions in breast cancer development and the clinical ramifications of these findings merit further investigation.

## RESULTS

### Effect of IGF1R or INSR abrogation on p53 expression and phosphorylation

An increasing body of experimental and clinical evidence has identified a direct link between obesity, hyperinsulinemia and cancer risk. The role of the INSR in the initiation and progression of cancer, in general, and breast cancer in particular, has been a controversial issue for many years [29, 30]. Likewise, the interplay between p53, an important player in cancer etiology, and the insulin pathway has not yet been unequivocally defined. To investigate the functional and physical interactions between INSR and p53 we employed MCF7 breast cancer-derived cells with specific disruptions of the INSR (INSR-KD) or IGF1R (IGF1R-KD) receptors [20]. The characterization of these cells has been recently reported [31]. Briefly, cells with a disrupted INSR or IGF1R did not express the abrogated receptor in neither cytoplasmic nor nuclear fractions. The existence of a compensatory mechanism was suggested by experiments showing enhanced IGF1R levels in both cytoplasmic and nuclear fractions of INSR-KD cells.

**Figure 1:**
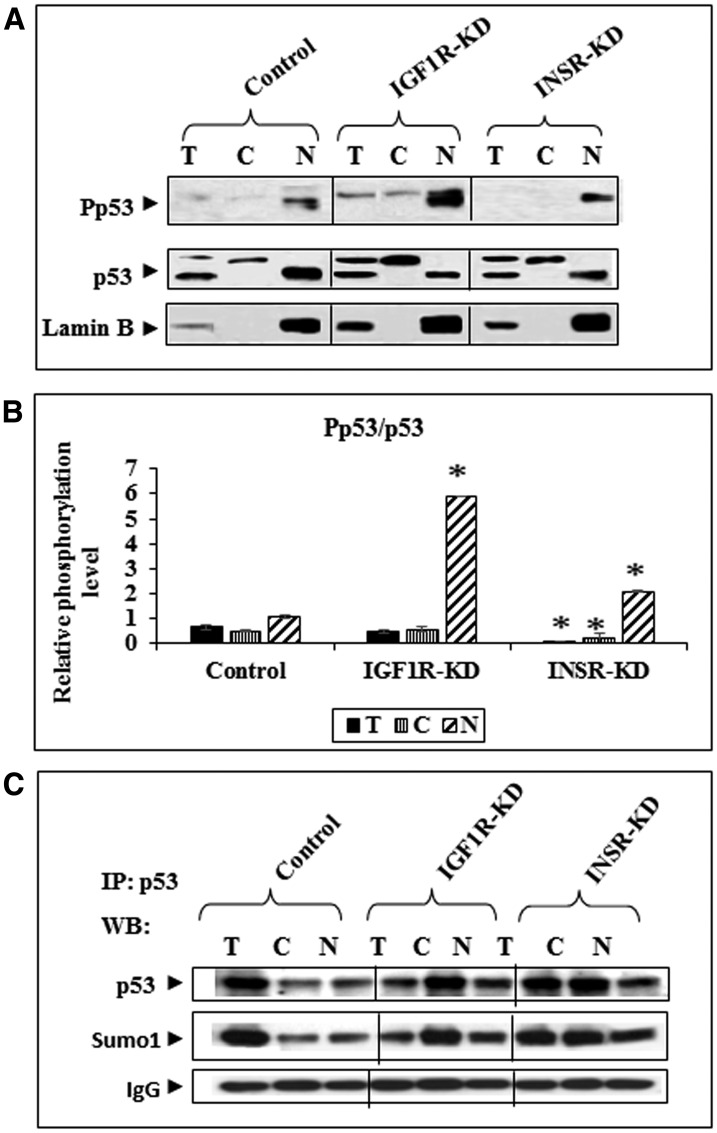
Western blot analysis of total and phospho-p53 in IGF1R-KD and INSR-KD cells. (**A**) Total (100 μg protein), cytoplasmic (40 μg) and nuclear (40 μg) fractions of IGF1R-KD, INSR-KD and control MCF7 cells were electrophoresed through SDS-PAGE gels, blotted onto nitrocellulose membranes and incubated with antibodies against total and phospho-p53. Lamin B was used as a nuclear marker. (**B**) Relative phosphorylation of p53 in total and subcellular fractions was calculated by normalizing phospho- to total p53 levels. Data represent two independent experiments (mean ± SEM; ^*^ *p* < 0.01 versus respective fraction in control cells). (**C**) Effect of IGF1R-KD or INSR-KD on p53 sumoylation. Total and subcellular fractions of IGF1R-KD, INSR-KD and control MCF7 cells were immunoprecipitated with anti-p53, electrophoresed and immunoblotted with a Sumo-1 antibody. IgG was used as a control for the co-IP experiment.

To evaluate the impact of insulin and IGF1 signaling on p53 expression and activation status, Western blots were conducted on total, cytoplasmic and nuclear fractions of control, IGF1R-KD and INSR-KD cells. No major differences in total p53 levels were seen between cell lines (Figure 1A, 1B). However, while the nuclear fractions contain mainly a ~53-kDa band, a larger p53 molecule was detected in the cytoplasmic fractions. The nature of this band was not investigated, although we assume that it corresponds to a modified p53 molecule. Both p53 bands were detected in whole cell extracts. Western blot analysis using an antibody against phospho-p53 detected higher phospho-p53 levels in cytoplasmic and, particularly, nuclear fractions of IGF1R-KD cells. This expression pattern suggests that activation of p53 is negatively regulated by IGF1R. Hence, absence of IGF1R expression in IGF1R-KD cells leads to relaxation of inhibitory control with an ensuing rebound in p53 phosphorylation.

In addition, we assessed the impact of IGF1R or INSR abrogation on p53 sumoylation, an important modification of the p53 molecule. To this end, total, cytoplasmic and nuclear extracts of IGF1R-KD, INSR-KD and control cells were immunoprecipitated with anti-p53, electrophoresed through SDS-PAGE and immunoblotted with anti-Sumo1. Results of IP experiments revealed a minor increase in sumoylated p53 in the cytoplasmic fractions of both disrupted, compared to control, cells (Figure 1C). These results indicate that IGF1R/INSR status does not have a major role on sumoylation (and, probably, degradation) of p53.

### Regulation of the INSR promoter by wild-type and mutant p53

**Figure 2:**
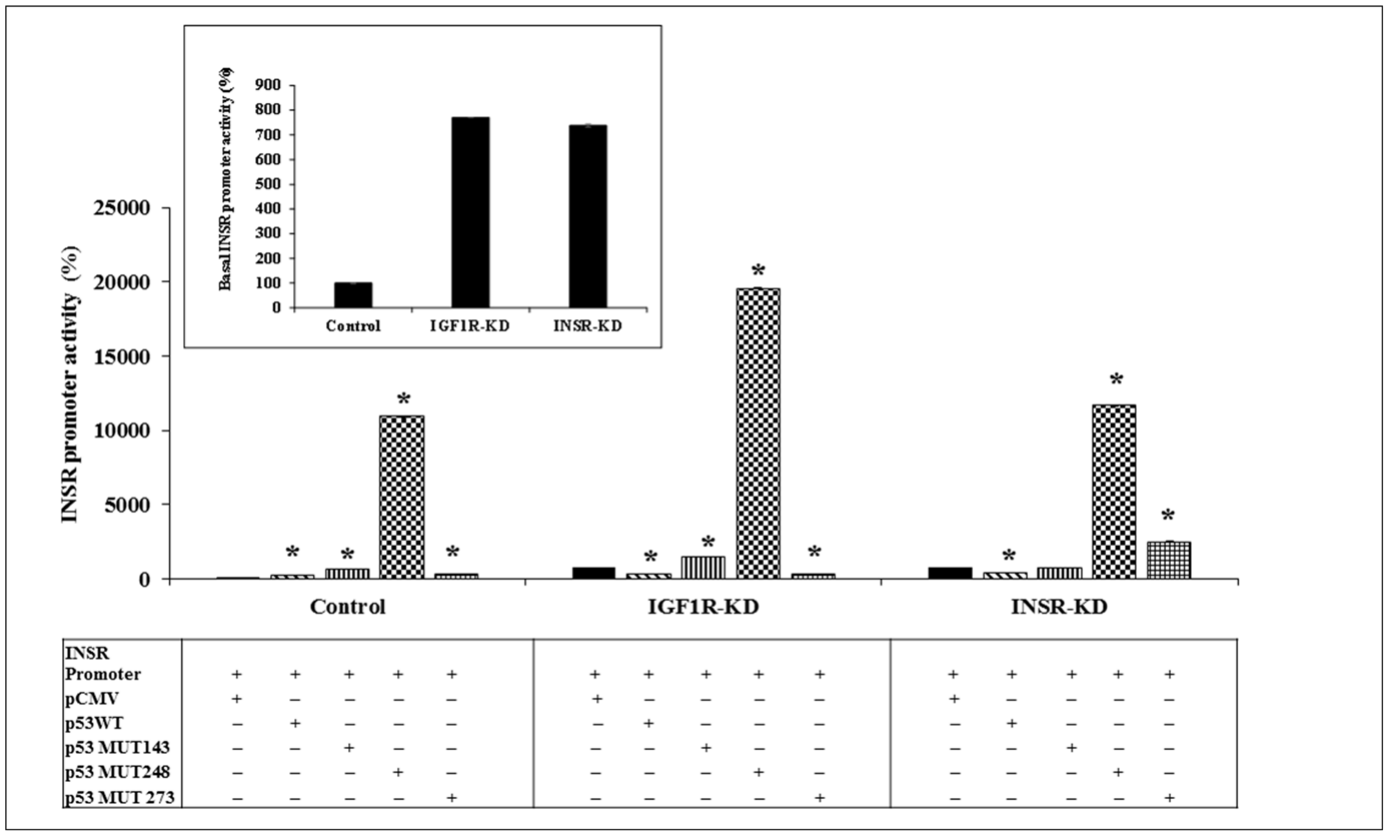
Effect of wild-type or mutant p53 on *INSR* promoter activity. IGF1R-KD, INSR-KD and control MCF7 cells were transiently co-transfected with the pGL3(-877/-2) LUC *INSR* promoter luciferase reporter along with a wild-type or mutant (MUT143, 248 or 273) p53-encoding expression vector. Cells were harvested after 48 h and luciferase activity was measured. The activity of the *INSR* promoter is expressed as luciferase values normalized to total protein. A value of 100% was assigned to the promoter activity generated by the *INSR* promoter construct in control cells. The inset presents the basal *INSR* promoter activity levels at a large scale. ^*^ *p* < 0.01 *versus* empty vector-transfected cells. Experiments were performed in triplicates.

Next, we investigated the regulation of the *INSR* promoter by wild-type or mutant p53. To this end, IGF1R-KD, INSR-KD and control cells were transfected with an *INSR* promoter-luciferase reporter construct [pGL3(-877/-2) LUC] along with expression vectors encoding either wild-type or mutant p53 (including mutations at codons 143, 248 or 273). Results of transfection assays indicate that basal *INSR* promoter activity was ~7-fold higher in IGF1R-KD and INSR-KD cells than in control cells (Figure 2). These results suggest that abrogation of either one of these receptors leads to relaxation of inhibition of the *INSR* promoter. In addition, cotransfection of a wild-type p53 expression vector inhibited *INSR* promoter activity by 57% and 44% in IGF1R-KD and INSR-KD cells, respectively. In contrast, wild-type p53 stimulated *INSR* promoter activity by 2.6-fold in control cells. These results indicate that intact INSR and/or IGF1R pathways are necessary in order for p53 to stimulate the *INSR* promoter. Abrogation of either one of these pathways results in an inhibitory effect of p53.

**Figure 3:**
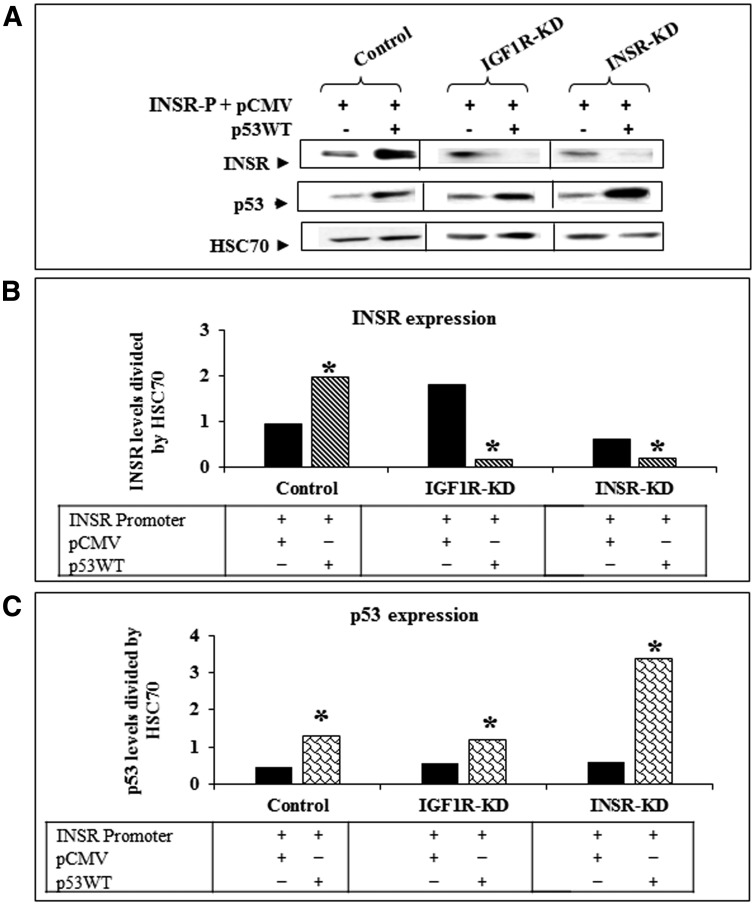
Effect of wild-type p53 transfection on INSR protein expression. (**A**) IGF1R-KD, INSR-KD and control MCF7 cells were co-transfected with an *INSR* promoter luciferase reporter along with a wild-type p53 expression vector, as described in the legend to Figure 3. After 48 h cells were harvested and the expression of the endogenous INSR was assessed by Western blots. HSC70 was measured as a loading marker. Panels (**B**) and (**C**) show scanning densitometric analyses of INSR and p53 protein levels, respectively, normalized to HSC70. ^*^ *p* < 0.01 *versus* empty vector-transfected cells.

To assess whether the changes in *INSR* promoter detected above are correlated with corresponding changes in INSR protein levels, Western blots were performed using whole cell extracts of wild-type p53-transfected (or control) cells. As shown in Figure 3, transfection of p53 enhanced endogenous INSR levels in control cells. On the other hand, exogenous p53 reduced INSR levels in IGF1R-KD and INSR-KD cells. These results indicate that the effect of p53 on transcription of the *INSR* gene is reflected in concomitant changes in expression levels of functional receptors.

Next, we explored the effect of mutant p53 on *INSR* promoter activity. Coexpression of p53MUT248 enhanced *INSR* promoter activity in all three cell lines, but with significant differences between control and disrupted cells (109-fold increase in control cells, 25-fold increase in IGF1R-KD cells and 15-fold increase in INSR-KD cells) (Figure 2). p53MUT143 enhanced *INSR* promoter activity by 6.6-fold in control cells and 2-fold in IGF1R-KD cells; no effect was seen in INSR-KD cells. Finally, p53MUT273 inhibited *INSR* promoter activity in IGF1R-KD cells while stimulating promoter activity in INSR-KD cells.

### Interactions between INSR/IGF1R and p53 in regulation of the INSR promoter

**Figure 4:**
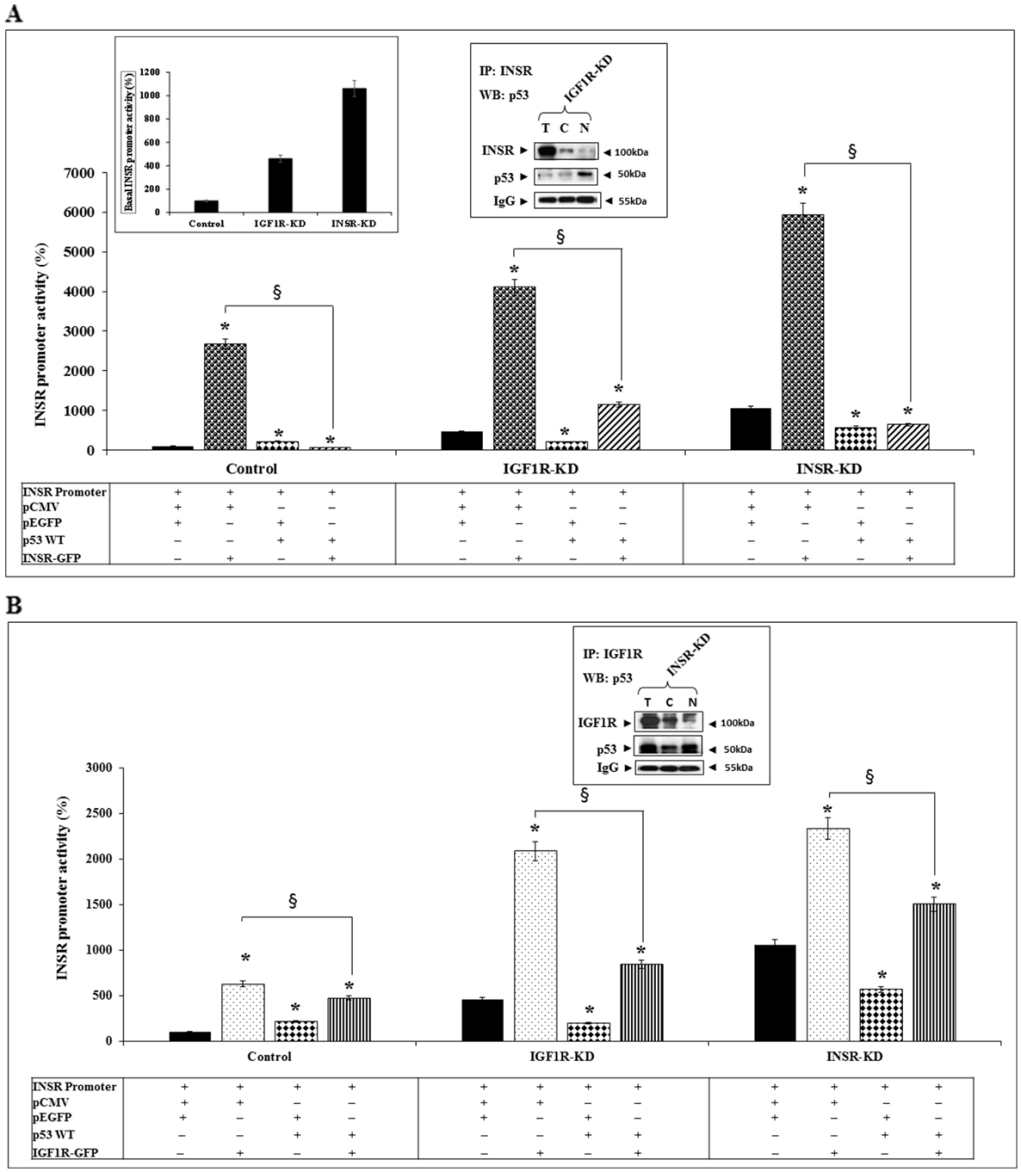
Functional interactions between INSR/IGF1R and p53 in regulation of *INSR* promoter activity. (**A**) IGF1R-KD, INSR-KD and control MCF7 cells were co-transfected with the pGL3(-877/-2) LUC *INSR* promoter luciferase reporter along with an INSR-A expression vector (INSR-GFP) (or empty pEGFP vector) and a wild-type p53 expression vector (or empty pCMV). After 48 h cells were harvested and *INSR* promoter activity was measured as described in the legend to Figure 2. The left inset presents the basal *INSR* promoter activity levels at a large scale. Total, cytoplasmic and nuclear fractions of IGF1R-KD cells were immunoprecipitated with an INSR antibody, electrophoresed and immunoblotted with anti-INSR and anti-p53 (right inset). IgG was used as a control for the co-IP experiment. ^*^ *p* < 0.01 *versus* control cells. ^§^ *p* < 0.01 *versus* empty pCMV-transfected cells. (**B**) Cells were transfected with the *INSR* promoter reporter along with an IGF1R expression vector (or empty pEGFP) and a p53 expression vector (or empty pCMV). Cells were processed as described above. Total, cytoplasmic and nuclear fractions of INSR-KD cells were immunoprecipitated with an IGF1R antibody, electrophoresed and immunoblotted with anti-IGF1R and anti-p53 (inset).

To investigate the potential functional interaction between p53 and INSR in regulation of the *INSR* gene, transient co-transfection assays were performed using an *INSR* promoter-luciferase reporter construct along with a wild-type p53 expression vector (or empty pCMV) and an INSR-A expression vector (or empty pEGFP). Results of cotransfection experiments demonstrate that INSR-A stimulated *INSR* promoter activity in all three cells lines but with major differences between control and disrupted cells. Thus, whereas exogenous INSR-A enhanced promoter activity by 27-fold in control cells, the stimulatory effects in IGF1R-KD and INSR-KD cells were 8.9- and 5.6-fold, respectively (Figure 4A). Coexpression of wild-type p53 abolished the stimulatory effect of INSR-A. Again, significant differences were seen between control (40-fold decrease) and KD (3.5- to 9.1-fold decreases) cells. Co-IP experiments revealed a physical interaction between INSR and p53 in nuclear fractions of IGF1R-KD cells (Figure 4A, right inset).

We then assessed the capacity of IGF1R to activate the *INSR* promoter, and the interactions between IGF1R and p53. Results of cotransfection experiments indicate that the ability of IGF1R to stimulate *INSR* promoter activity was 40-50% lower than that of INSR-A in all three cell lines (Figure 4B). Results of co-IP assays in INSR-KD cells identified a protein-protein interaction between IGF1R and p53 in both cytoplasmic and nuclear fractions (Figure 4B, inset). Taken together, data indicate that both INSR and IGF1R are capable of stimulating *INSR* promoter activity, although the cognate receptor exhibited a stronger effect. In addition, we identified a physical interaction between p53 and INSR/IGF1R that might explain the ability of p53 to reduce the stimulatory effects of both INSR and IGF1R.

### Physical interactions between p53, INSR/IGF1R and the INSR promoter

**Figure 5:**
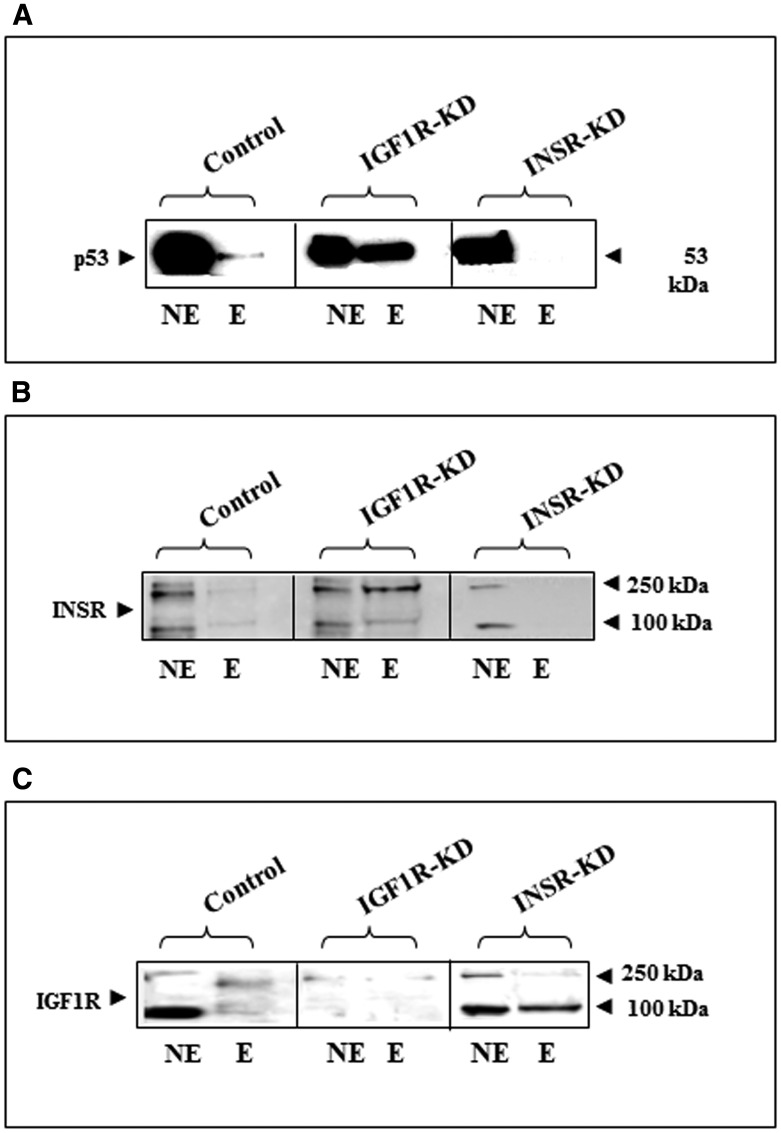
Physical interactions between p53, INSR/IGF1R and the *INSR* promoter region. (**A**) A genomic fragment extending from nt -540 to -18 of the *INS*R promoter was labeled using a 5′-biotinylated antisense primer. The labeled fragment was bound to streptavidin magnetic beads, incubated with nuclear extracts of IGF1R-KD, INSR-KD or control cells, and eluted with a high-salt buffer. Western blots were performed on nuclear extracts (NE) and eluted material (E) using a p53 antibody. DNA affinity chromatography was also employed to assess the binding of endogenous INSR protein (**B**) and endogenous IGF1R protein (**C**) to the *INSR* promoter region.

To assess the potential binding of p53 to the *INSR* promoter, a genomic fragment extending from nt -540 to -18 (relative to the translation start site) was labeled using a 5′-biotinylated primer. The labeled fragment was bound to streptavidin magnetic beads, incubated with nuclear extracts of IGF1R-KD, INSR-KD or control cells, and eluted with a high-salt buffer. Western blots using a p53 antibody identified a strong binding of p53 to the *INSR* promoter in IGF1R-KD cells (Figure 5A). These results suggest that diminished IGF1R levels in these cells might facilitate the direct binding of p53 to the proximal *INSR* promoter region. In addition, the fact that p53 did not bind at all to the *INSR* promoter in INSR-KD cells suggests that the presence of the INSR protein is important for p53 binding to the *INSR* promoter.

A similar approach was employed to investigate the binding of INSR and IGF1R proteins to the *INSR* promoter sequence. DNA affinity chromatography experiments showed that INSR displays a strong binding to the *INSR* promoter in IGF1R-KD cells (Figure 5B) whereas IGF1R exhibits a strong binding in INSR-KD cells (Figure 5C). Combined, these results suggest that lack of IGF1R in IGF1R-KD cells facilitates INSR binding to the promoter region. On the other hand, lack of INSR in INSR-KD cells facilitates IGF1R binding to the same sequences. Of notice, while the mature IGF1R (~100-kDa band) is mainly involved in *INSR* promoter binding, the precursor form of INSR (~250-kDa band) is predominantly associated with the *INSR* promoter region.

### Effect of INSR/IGF1R abrogation on the stimulatory effect of Sp1

**Figure 6:**
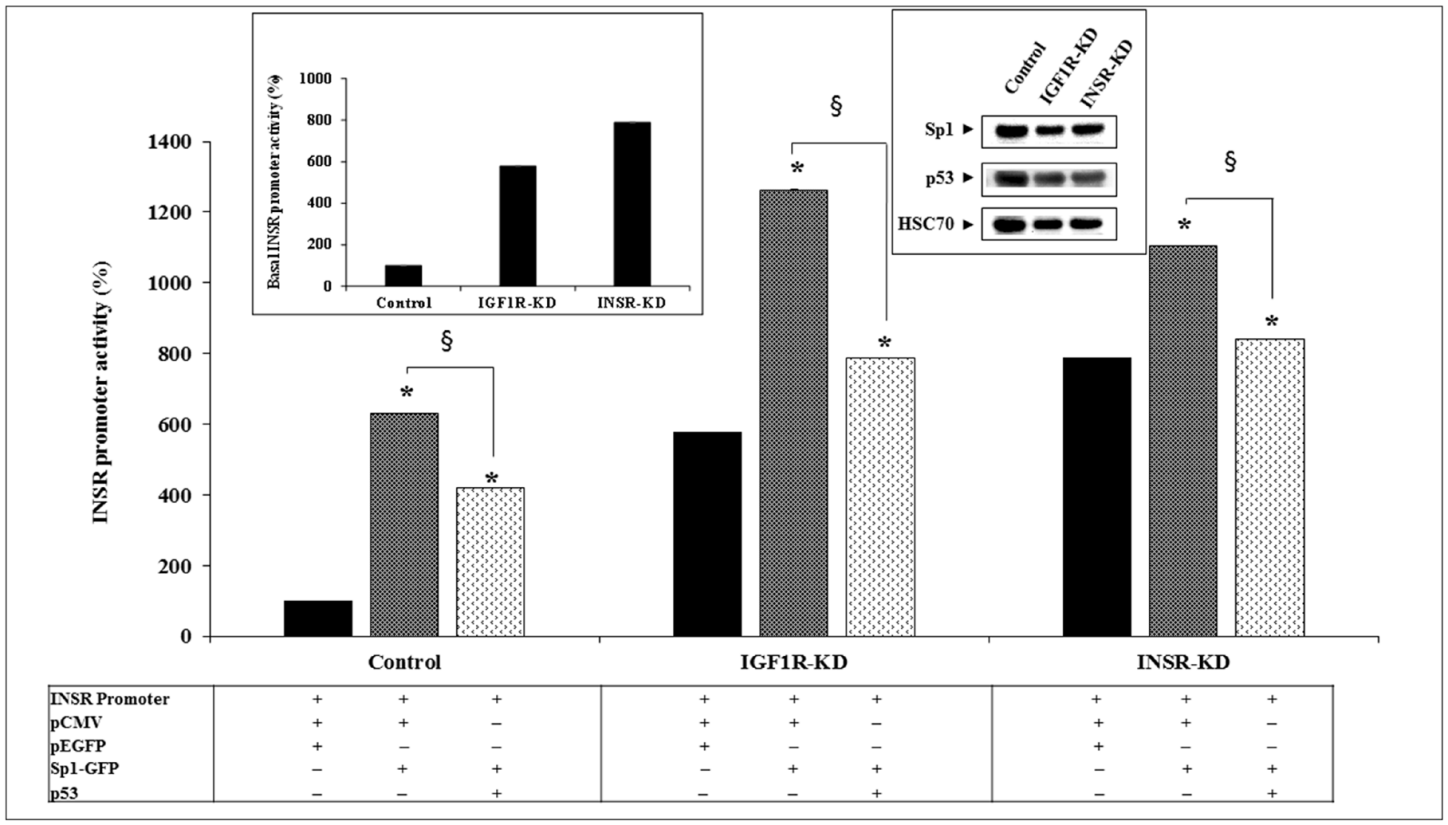
Effect of Sp1 on *INSR* promoter activity. IGF1R-KD, INSR-KD and control MCF7 cells were transiently co-transfected with the pGL3(-877/-2) LUC *INSR* promoter luciferase reporter along an Sp1 expression vector (or empty pEGFP plasmid) and a wild-type p53 vector (or empty pCMV). Cells were harvested after 48 h and promoter activity was measured. ^*^ *p* < 0.01 *versus* control cells. ^§^ *p* < 0.01 *versus* empty pCMV-transfected cells. The left inset presents the basal *INSR* promoter activity levels in all three cell lines at a large scale. The right inset presents the endogenous levels of Sp1 and p53 proteins in control and KD cells.

Transcription factor Sp1 has been identified as a key activator of the *INSR* gene [32]. To assess the impact of INSR or IGF1R silencing on the ability of Sp1 to transactivate the *INSR* promoter, cotransfection assays were conducted using an *INSR* promoter construct along with an Sp1 expression vector. Transfection assays indicate that the ability of Sp1 to activate the *INSR* promoter was impaired in IGF1R-KD and INSR-KD cells (Figure 6). Thus, whereas Sp1 enhanced *INSR* promoter activity by 6.3-fold in control cells, it only stimulated promoter activity by ~1.4-2.2-fold in disrupted cells. Co-expression of p53 diminished the stimulatory effect of Sp1 in all of the cell lines.

### Effect of INSR/IGF1R abrogation on the effect of p53 on cell proliferation

**Figure 7:**
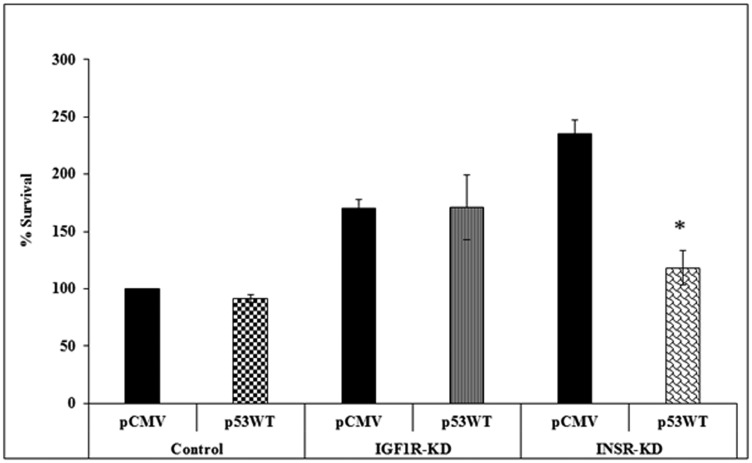
Effect of p53 on cell proliferation. IGF1R-KD, INSR-KD and control MCF7 cells were plated in 96-well plates in quadruplicate and, after 24 h, were transfected with p53-WT or empty vector (pCMV). Proliferation was measured after an additional 72 h using an XTT assay. A value of 100% was given to the cell number of control, untreated cells at the end of the incubation period. ^*^ *p* < 0.01 *versus* respective pCMV-transfected cells.

To investigate the impact of INSR or IGF1R abrogation on the effect of p53 on cell proliferation, IGF1R-KD, INSR-KD and control cells were transfected with a p53 expression vector in serum-containing media, and proliferation was assessed after 72 h using an XTT assay. Results obtained indicate that, under basal conditions, proliferation was enhanced in both KD cells (Figure 7). These results might imply that abrogation of a specific receptor leads to compensatory expression of the opposite receptor, with ensuing increase in proliferation. This effect was particularly evident in INSR-KD cells that express high levels of IGF1R. Expression of p53 diminished proliferation by ~50% in INSR-KD, but not IGF1R or control, cells.

### Effect of INSR/IGF1R abrogation on p53-stimulated cell cycle dynamics

**Figure 8:**
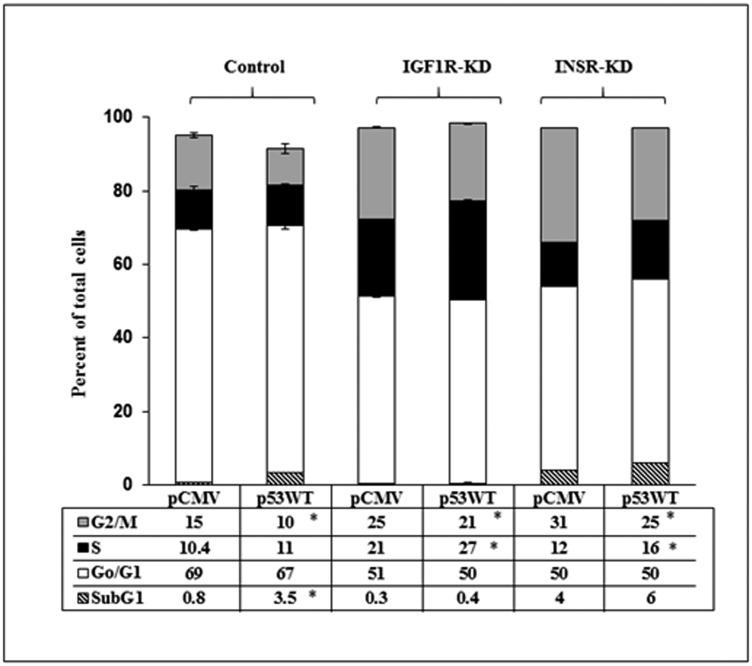
Effect of p53 on cell cycle dynamics. IGF1R-KD, INSR-KD and control MCF7 cells were seeded in triplicate onto 6-well plates and, after 24 h, were transfected with p53-WT (or empty pCMV vector). After an additional 24 h, the cells were tripsinized, counted and plated again (in 6-well plates in triplicate, 10^5^ cells/well) for 72 h. Cells were then permeabilized with Triton X-100, stained with propidium iodide and analyzed using a FacsCalibur system. ^*^ *p* < 0.01 *versus* respective pCMV-transfected cells.

Finally, cell cycle progression was assessed in INSR/IGF1R-disrupted cells expressing (or lacking) a wild-type p53. Under basal conditions, major increases were seen in the portion of cells at G2/M in both KD, in comparison to control, cells (Figure 8). p53 expression led to a 33% decrease in control cells at this stage, while the effect of p53 was attenuated in IGF1R-KD (16% decrease) and INSR-KD (22% decrease) cells. Finally, whereas p53 expression had no effect on the proportion of control cells at the S phase, a marked increase in the portion of cells at this stage was seen in IGF1R-KD (28% increase) and INSR-KD (33% increase) cells. Finally, the negative impact of p53 transfection on INSR-KD cells proliferation, described in the previous section, cannot be explained by the results of cell cycle analysis described here. Differences in the sensitivities and end-points between XTT and cell cycle assays may probably explain this discrepancy.

## DISCUSSION

INSR overexpression is regarded as a common feature of many types of cancer [16, 33]. In the vast majority of INSR-expressing tumors, the A-isoform (lacking exon 11) predominates over the B-isoform [30]. While the relative contribution of the INSR and IGF1R signaling pathways to the development of a malignant phenotype has been a controversial issue for many years, a mounting volume of experimental, clinical and epidemiological data provides strong support to the notion that insulin signaling is central to cancer etiology [34]. Among other lines of evidence, the connection between obesity, hyperinsulinemia and enhanced cancer risk provides a biologically plausible rationale for a key role of insulin and the INSR in breast cancer initiation and/or progression.

Accumulation of mutations constitutes an early event in cellular transformation and may, eventually, lead to the establishment of a cancerous phenotype. P53-mediated cell cycle arrest enables damaged DNA to be repaired before the replicative phase of the cell cycle [25, 26, 35, 36]. Alternatively, p53 can elicit an apoptotic program. The present study was aimed at evaluating the hypothesis that tumor suppressor p53 governs the expression and activation of the *INSR* gene in breast cancer (and, probably, other) cells. In addition, the study was designed to investigate the mechanism of action of p53 and the physical interactions between p53 and the *INSR* gene. Finally, using breast cancer-derived cell lines with disruption of either the IGF1R or INSR we assessed the specific impact of the IGF1R and INSR pathways on p53 expression and activity.

Cellular fractionation experiments revealed enhanced p53 phosphorylation in nuclear fractions of IGF1R-KD, but not INSR-KD, cells. These results suggest that activation of p53 is predominantly inhibited by IGF1R, while the INSR pathway has a minor effect on p53 phosphorylation. Furthermore, results of transient cotransfection assays indicate that: (1) INSR promoter activity was markedly increased (~7-fold) in cells with disrupted IGF1R or INSR; and (2) wild-type p53 inhibited *INSR* promoter activity by 57% and 44% in IGF1R-KD and INSR-KD cells, respectively, but not in cells with intact IGF1R/INSR pathways. Taken together, data indicate that the *INSR* promoter is under constitutive inhibition by both IGF1R and INSR. Consequently, silencing of either one of these receptors leads to relaxation of inhibition. In addition, functional INSR or IGF1R pathways are needed in order for wild-type p53 to stimulate the *INSR* promoter. Abrogation of either one of these pathways leads to an inhibitory effect of p53.

In terms of the mechanism of action of p53 in regulation of the *INSR* promoter, DNA affinity chromatography experiments showed a strong binding of p53 to the *INSR* promoter region in IGF1R-KD cells. This pattern is consistent with the idea that reduced IGF1R concentrations in IGF1R-KD cells might promote the direct binding of p53 to the *INSR* promoter region. Likewise, this approach identified a direct binding of INSR and IGF1R proteins to the *INSR* promoter sequence in IGF1R-KD and INSR-KD cells, respectively. In summary, the ability of p53 to govern *INSR* gene transcription is dictated by complex physical (protein-protein and protein-DNA) interactions involving, in addition to p53, also the INSR and IGF1R proteins. The ability of both receptors to translocate to the cell nucleus and to display DNA binding capacities has been recently reported [37–41].

We and others have previously reported that p53 interacts with the IGF pathway at a number of levels, including transcriptional regulation of IGF axis components (i. e., *IGF1R, IGF2 and IGFBP3* genes) [27, 42–44]. The IGF1 and p53 signaling pathways were shown to converge in both cytoplasmic and nuclear compartments in a bidirectional fashion [45]. Hence, whereas p53 governs IGF system expression and activation, IGF1 regulates the p53/p63/p73 system [46]. *Gain-of-function* mutations of *p53* in cancer disrupts its inhibitory activity and generates oncogenic molecules capable of transactivating the *IGF1R* gene. The capacity of p53 to regulate the *INSR* promoter was originally published by Webster et al. [47]. The authors reported a correlation between INSR and p53 expression in breast tumors. Moreover, exogenous p53 was able to repress the *INSR* promoter whereas a dominant-negative p53 (p53MUT248) derepressed the promoter in cells with normal p53. In addition, authors provided evidence that the effect of p53 was mediated by C/EBP and Spl transcription factors. Data presented here demonstrate that the ability of Sp1 to activate the *INSR* promoter was impaired in IGF1R-KD and INSR-KD cells. The stimulatory effect of Sp1 was reduced by p53 co-expression. The main difference between the Webster et al. paper and the present study is the fact that different, though overlapping, *INSR* promoter fragments were used. Thus, Webster et al. employed a ~1.5-kb fragment extending from nt -290 to -1823 of the hINSR promoter [47] whereas our construct extended from nt -2 to -877. As described above, we show that wild-type p53 enhanced promoter activity in control, but not IGF1R/INSR disrupted, cells.

Evidence accumulated in recent years indicate that, in addition to its capacity to govern cell cycle progression, p53 activation has also a major impact on metabolic processes, including glucose transport [48] and obesity [49]. p53 was shown to bind to more than 4,000 sites in the human genome and to regulate the expression of several hundred genes [50]. Among these genes, critical regulators of glucose, lipids and amino acids metabolism were identified. Moreover, p53 emerged as an important player in control of oxidative phosphorylation and reactive oxygen species generation. It has been suggested that induction of p53 expression may induce senescence, autophagy and apoptosis, which are tightly linked to the PI3K/Akt/mTOR pathway. Our data, linking p53 expression and action to the INSR axis, may be of major relevance in efforts to understand the role of p53 and insulin in regulation of cancer cell metabolism.

In conclusion, we have presented evidence that the *INSR* gene constitutes a downstream target for p53 action. Whereas wild-type p53 stimulated *INSR* promoter activity in control MCF7 cells, disruption of endogenous IGF1R or INSR led to inhibition of promoter activity by wild-type p53. Mutant, oncogenic versions of p53, for the most part, strongly stimulated *INSR* promoter. In addition, p53 exhibits direct binding to the *INSR* promoter region in cells with a disrupted IGF1R. Taken together, data presented here identifies complex functional and physical interactions between p53 and the INSR pathway. The clinical implications of this interplay in breast cancer needs to be critically assessed.

## MATERIALS AND METHODS

### MCF7 breast cancer-derived INSR-KD and IGF1R-KD cell lines

The generation of MCF7-derived INSR-knock down (KD), IGF1R-KD and control (non-coding control shRNA sequence) cell lines has been recently described [20]. Cell lines were provided by Drs. Derek LeRoith and Ran Rostoker (Technion, Haifa, Israel). MCF7-derived stable cell lines were maintained in DMEM supplemented with 10% fetal bovine serum, 100 units/ml penicillin, 100 μg/ml streptomycin, 5.6 mg/l amphotericin B, and 1 μg/ml puromycin. In some experiments, cells were treated with IGF1 (PeproTech Ltd., Rocky Hill, NJ, USA) or insulin (Biological Industries Ltd., Bet-Haemek, Israel) at a dose of 50 ng/ml. All experiments were carried out at least twice.

### PCR and DNA affinity chromatography of the INSR promoter

For DNA affinity chromatography, a 523-bp human proximal *INSR* promoter fragment extending from nucleotides -540 to -18 was labeled using a 5′-biotinylated antisense primer, as described previously [51]. This fragment includes most of the proximal 5′-flanking region and three initiator sites located 276, 282 and 283 bp upstream of the translation site. Primer sequences were derived from genomic *INSR* [52] as follows: sense, 5′-GTCTCCTCGGATCAGAGCGC-3′; antisense, 5′-(Biotin)-GAGTCCCTTCCTAGGCCAGATC-3′. PCR was performed using the TermalAce^™^ DNA Polymerase reagent (InVitrogen, Carlsbad, CA, USA). The biotinylated PCR product was bound to streptavidin magnetic beads (Dynabeads^®^ M-270 Streptavidin; Dynak Biotech ASA, Oslo, Norway), and incubated with nuclear extracts of IGF1R-KD, INSR-KD or control cells. *INSR* promoter-binding proteins were eluted with a high salt-containing buffer and analyzed by Western blots as described below.

### Protein analysis and immunoblotting

Total cell lysates and cytosolic and nuclear extracts were prepared as described [31]. Samples were electrophoresed through 10% SDS–PAGE, followed by transfer to nitrocellulose membranes. Blots were blocked with 5% skim milk and incubated overnight with antibodies listed below. Antibodies against IGF1R β-subunit (#3027), insulin receptor β-subunit (#3025), phospho-p53 (#9284) and SUMO-1 (#5718) were from Cell Signaling Technology (Danvers, MA, USA). Antibodies against p53 (mixture: DO-1 and 1801) and heat shock cognate HSC70 (#B-6) were from Santa Cruz Biotechnology (Dallas, TX, USA). An antibody against lamin B was from Abcam (Cambridge, UK). Blots were washed and incubated with the appropriate horseradish peroxidase-conjugated secondary antibody. Protein A-peroxidase conjugated was used for p53 detection (Rockland Immunochemicals Inc, Limerick, PA, USA). Proteins were detected using the enhanced chemiluminiscence reaction (Westar Supernova, Cyanagen, Bologna, Italy). HSC70 was used as a loading control and lamin B as a nuclear marker.

### Co-immunoprecipitation (co-IP) assays

Total (500 μg), cytoplasmic (100 μg) and nuclear (100 μg) extracts were diluted 1:2 with IP dilution buffer [1% Triton X-100, 150 mM NaCl and 20 mM Tris buffer (pH 7.5) containing proteases and phosphatases inhibitors], and immunoprecipitated with anti-p53, anti-IGF1R β-subunit or anti-INSR β-subunit overnight at 4°C. Protein A/G-agarose beads (SC-20003; Santa Cruz Biotechnology) were added for 2 h. Sample buffer was added to the samples and electrophoresed through 10% SDS-PAGE. Nitrocellulose membranes were blotted with anti-p53, anti-SUMO-1, anti-IGF1R β-subunit or anti-INSR β-subunit, as described above.

### Plasmids and transient co-transfections

For transient co-transfection experiments, an *INS*R promoter-luciferase reporter construct extending from position -877 to -2 of the 5′-untranslated region of the human *INS*R gene [pGL3 (−877/–2) luciferase (LUC) vector] was employed. The plasmid was kindly provided by Dr. Antonino Belfiore (University of Catania, Italy). Expression vectors encoding wild-type and mutant p53 were provided by Dr. Edward Mercer (Thomas Jefferson University, Philadelphia, PA, USA). A wild-type p53 (p53WT) expression vector was constructed into the pCMV-Neo-Bam vector. p53MUT143 encodes p53 harboring a Val to Ala mutation at position 143. p53MUT248 contains an Arg to Trp mutation at position 248 and p53MUT273 is a mutant p53 in which an Arg residue at position 273 was mutated to His. All three mutants were cloned in the pCMV-Neo-Bam expression vector as described [53]. A GFP-IGF1R expression plasmid was provided by Dr. Rosemary O’Connor (University of Cork, Ireland) [54]. An INSR isoform-A expression vector was a gift from Dr. Antonino Belfiore [55]. For Sp1 expression experiments, the pEGFP-Sp1 vector was employed (Addgene, Watertown, MA, USA). For co-transfection experiments, cells were transfected with 1 μg of the *INSR* promoter reporter along with 1 μg of wild-type or mutant p53 (or empty pCMV vector). In certain experiments, cells were co-transfected with 1 μg of expression vectors encoding IGF1R or INSR-A (GFP-IGF1R or GFP-INSR-A) or Sp1 expression vector. Transfections were carried out using the Jet-PEI transfection reagent (Polyplus Transfection Inc, Illkirch, France). Cells were harvested 48 h after transfection, and luciferase activity was measured as described [31]. Promoter activities are expressed as luciferase values normalized to protein concentrations. Bars represent the mean ± SEM of relative luciferase activity from triplicate samples in two separate experiments.

### Proliferation assays

Cells were seeded in quadruplicate onto 96-well plates (2 × 10^3^ cells/well) and, after 24 h, were transfected with p53-WT or empty vector (pCMV). Cell proliferation was determined by an XTT cell proliferation kit (Biological Industries) after 72 h. The colorimetric reaction was measured using an ELISA reader at a wavelength of 450 nm and a reference absorbance of 630 nm in at least three independent assays. Cell proliferation was expressed as a percentage of optical density values obtained in p53-WT-transfected cells relative to empty vector-transfected cells. All experiments were performed in quadruplicate in three independent experiments.

### Cell cycle analysis

Cells were seeded in triplicate onto 6-well plates. After 24 h the cells were transfected with p53-WT or empty vector, as described above. After 24 h, the cells were tripsinized, counted and plated again (in 6-well plates in triplicate, 10^5^ cells/well) for 72 h. After incubation, the cells were washed with phosphate-buffered saline (PBS), trypsinized, centrifuged and resuspended in PBS. The cells were permeabilized with Triton X-100 before adding propidium iodide. Stained cells were analyzed using a FacsCalibur system (Cytek Development Inc, Fremont, CA, USA).

### Statistical analyses

The statistical significance of the differences between groups was assessed by Student’s *t* test (two samples, equal variance). Scanning densitometry analyses were evaluated using TINA imaging analysis software. Signal intensities of proteins were normalized to the corresponding HSC70 protein signals. Data are presented as mean ± SEM of three independent experiments. *P* values < 0.05 or < 0.01 were considered statistically significant.
